# In Vitro Restoration of Colistin Susceptibility by Ivacaftor Synergy with Limited Reproducibility in a Murine Pneumonia Model

**DOI:** 10.3390/antibiotics15040414

**Published:** 2026-04-18

**Authors:** Ana Verónica Halperin, Franziska Schwartz, Lars Christophersen, José Pérez-del Palacio, Manuel Ponce-Alonso, José Avendaño-Ortiz, Juan de Dios Caballero, Rafael Cantón, Claus Moser, Rosa del Campo

**Affiliations:** 1 Servicio de Microbiologia, Hospital Universitario Ramón y Cajal and Instituto Ramón y Cajal de Investigación Sanitaria (IRYCIS), 28034 Madrid, Spain; ana.halperin@gmail.com (A.V.H.);; 2 Department of Clinical Microbiology, Copenhagen University Hospital, 2100 Copenhagen, Denmark; 3 Fundación MEDINA, Centro de Excelencia en Investigación de Medicamentos Innovadores en Andalucía, Armilla, 18016 Granada, Spain; 4 CIBER de Enfermedades Infecciosas, Instituto de Salud Carlos III, 28029 Madrid, Spain; 5 Department of Immunology and Microbiology, Copenhagen University, 2200 Copenhagen, Denmark; 6 Facultad de Ciencias de la Salud, Universidad de La Rioja, 26006 Logroño, Spain

**Keywords:** ivacaftor, colistin resistance, in vivo synergy, phosphoethanolamine, *Pseudomonas aeruginosa*, *Klebsiella pneumoniae*

## Abstract

**Background:** We aimed to investigate the potential synergistic effect of ivacaftor combined with colistin against *Pseudomonas aeruginosa* and *Klebsiella pneumoniae*, and to elucidate the underlying molecular mechanisms through metabolomic analysis and its reproducibility in a murine model. **Methods:** Six colistin-susceptible and 2 colistin-resistant cystic fibrosis *P. aeruginosa* isolates, along with two colistin-resistant *K. pneumoniae* clinical isolates, were studied. Antimicrobial susceptibility was assessed by broth microdilution, and synergy by checkerboard assay. Metabolomic profiling was conducted via LC-HRMS with statistical analysis. A murine pneumonia model, induced by intranasal administration of colistin-resistant strains, was used to validate in vivo ivacaftor and colistin synergy after 24 h. **Results:** No previously described colistin resistance mutations were identified in *P. aeruginosa* strains, whereas *K. pneumoniae* carried *mgrB* variations. Ivacaftor restored colistin susceptibility at 16 mg/L concentration, and at 1–2 mg/L led to at least a twofold reduction in colistin MIC. Metabolomic analysis of colistin-resistant *P. aeruginosa* strains revealed that ivacaftor induced modifications in phosphoethanolamine groups of lipid A. However, no synergistic effects were observed in the short-term in vivo pneumonia model, regardless of the administration route. **Conclusions:** Ivacaftor exhibited no direct antimicrobial activity against *P. aeruginosa* and *K. pneumoniae* isolates in vitro but restored colistin susceptibility through synergistic interactions. The lack of synergy in the murine pneumonia model may reflect treatment time and challenges in standardizing in vivo conditions. These findings highlight the potential of ivacaftor as an adjunct to colistin therapy, warranting further investigation into its clinical applicability.

## 1. Introduction

Progressive pulmonary impairment due to chronic *Pseudomonas aeruginosa* colonization has historically been the leading cause of mortality in individuals with cystic fibrosis (CF). Recent advancements in CF therapeutics, particularly CF transmembrane conductance regulator (CFTR) modulators, have significantly altered the disease’s natural history [1]. These modulators include potentiators, such as ivacaftor, which enhance chloride channel opening, and correctors, such as lumacaftor, tezacaftor (first-generation), and elexacaftor (second-generation), which promote proper CFTR folding and trafficking to the apical cell membrane. Correctors are typically administered in combination with ivacaftor to optimize channel function, with the triple combination of ivacaftor, tezacaftor, and elexacaftor (Kaftrio^®^, Vertex Company, Boston, MA, USA) demonstrating superior efficacy. These treatments improve quality of life, stabilize lung function, and markedly reduce pulmonary infectious exacerbations [2].

An antimicrobial effect of ivacaftor has been proposed, evidenced by reduced *P. aeruginosa* density in the CF lungs [3,4,5]. However, it remains unclear whether this reduction results from ivacaftor’s direct antimicrobial activity or enhanced lung clearance due to improved CFTR function [6,7]. In vitro studies have reported ivacaftor’s bacteriostatic activity against *Staphylococcus aureus* and *Streptococcus* spp., but no direct effect against *P. aeruginosa* [8]. Notably, synergistic activity between ivacaftor and polymyxin B against *P. aeruginosa* has been observed in vitro, potentially linked to alterations in bacterial glycerophospholipid and fatty acid metabolism [9,10].

Polymyxins, including polymyxin B and polymyxin E (colistin), are amphipathic cationic lipopeptides used as last-resort antibiotics for multidrug-resistant Gram-negative infections, including CF-associated *P. aeruginosa* [11,12]. Despite similar structures, mechanisms of action, and antimicrobial spectra, polymyxins differ in pharmacokinetics and toxicity. Colistin is available as colistin sulfate for oral or topical administration and as colistimethate sodium for parenteral or inhaled delivery in CF [13]. Colistin’s antibacterial activity arises from its interaction with the anionic lipid A component of Gram-negative bacterial lipopolysaccharide (LPS), displacing the stabilizing Ca^2+^ and Mg^2+^ cations and disrupting membrane integrity [14,15]. Colistin resistance primarily results from mutations in the PhoP/PhoQ regulatory system (*mgr* genes), which reduce drug affinity by adding positive charges to lipid A [16]. Plasmid-mediated *mcr* genes, associated with resistance in Enterobacteriaceae, have not been reported in CF-associated *P. aeruginosa*. Despite chronic colistimethate sodium use in CF for exacerbation management, colistin resistance remains relatively uncommon [17].

The potential synergy between colistin and ivacaftor could enhance CF management and offer novel therapeutic options for severe infections, such as sepsis or pneumonia caused by colistin-resistant strains. This study aimed to evaluate the in vitro antimicrobial effects of ivacaftor, alone or in combination with colistin, against colistin-resistant *P. aeruginosa* and *Klebsiella pneumoniae* clinical isolates, with further validation in a murine model of acute pneumonia.

## 2. Results

### 2.1. Whole-Genome Sequence Analysis

The isolates used in this study were genetically unrelated and assigned to distinct sequence types (STs) (Table 1). Bioinformatic analysis did not identify known colistin resistance-associated genes or point mutations in either colistin-resistant or colistin-susceptible *P. aeruginosa* isolates. In contrast, mutations in the *mgrB* gene were detected in the colistin-resistant *K. pneumoniae* strains.

### 2.2. Antimicrobial Effects of CFTR Modulators Alone and in Combination with Colistin

No antimicrobial activity was observed for CFTR modulators (ivacaftor or lumacaftor) alone at concentrations up to 32 mg/L across all isolates. Colistin MICs for *P. aeruginosa* and *K. pneumoniae* isolates, alone and in combination with ivacaftor, lumacaftor, or both, are presented in Table 1. Notably, ivacaftor restored colistin-susceptibility in both colistin-resistant *P. aeruginosa* isolates (Pa1013 and Pa2713), reducing colistin MICs from ≥64 to 1 mg/L and from ≥64 to 2 mg/L, respectively. In colistin-susceptible *P. aeruginosa* isolates, ivacaftor caused a modest reduction in colistin MICs, which could be irrelevant and due to the accepted accuracy of ±one two fold dilution. Lumacaftor had no effect on colistin MICs in susceptible isolates but reduced MICs in colistin-resistant Pa1013 and Pa2713 from 64 to 8 mg/L, although this reduction did not reach the susceptibility breakpoint. The triple combination of colistin, ivacaftor, and lumacaftor showed no additional effect beyond that of colistin with ivacaftor alone. Similar results were observed for the two colistin-resistant *K. pneumoniae* isolates, with a less pronounced effect in the *Escherichia coli* NCTC 13846 (mcr-1-positive) control strain (Table 1).

The checkerboard assay was performed to explore the synergy between colistin (original MIC value) and ivacaftor (ranging from 0 to 16 mg/L) (Table 2). The results show that a concentration of ivacaftor between 1 and 2 mg/L was required to elicit at least 50% or more reduction in the MIC value of colistin. When combined with ivacaftor at 16 mg/L, the colistin MIC of all isolates was reduced by at least 4 serial dilutions.

### 2.3. Bacterial Metabolomic Analysis and Identification of Marker Compounds

**Figure 1:**
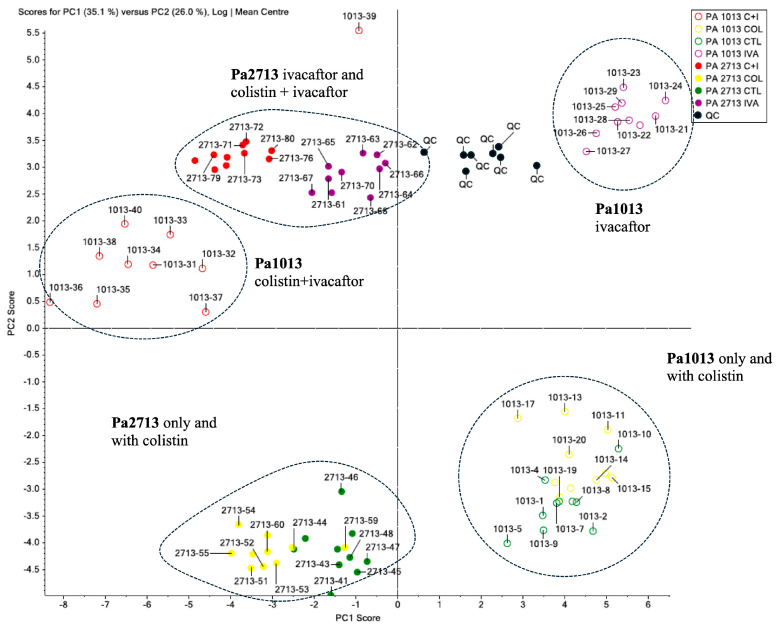
Principal component analysis of chromatograms obtained using positive ionization with the inoculum growth of Pa1013 and Pa2713 isolates in four different conditions: isolate without supplementation (control), supplementation with ivacaftor, with colistin, and with ivacaftor plus colistin. Green dots (quality controls, QC) represent the internal standard controls that ensure the quality and reproducibility of the method. The controls should be placed in the same quadrant of the PCA and as close together as possible to validate the method’s reproducibility, as shown in the figure.

Metabolomic analysis was performed on the colistin-resistant *P. aeruginosa* isolates Pa1013 and Pa2713. From 112 aliquots (2 strains, 4 conditions, 10 replicates per condition, 10 blanks, 10 internal controls, and 12 organic solvent aliquots), 2245 peaks were identified. Six peaks were statistically significant and differentially expressed following exposure to ivacaftor, colistin, or their combination (Figure 1). Five peaks corresponded to phosphoethanolamine molecules, and one was associated with *quorum-sensing* communication molecules (Table 3).

Both colistin-resistant *P. aeruginosa* isolates exhibited distinct lipid A modifications after culture with colistin, ivacaftor, or both drugs, with strain-specific differences observed. As expected, colistin supplementation did not significantly alter lipid A structure in these resistant isolates. However, each strain displayed unique lipid A compositions, potentially reflecting divergent strategies to evade colistin binding, as evidenced by their separation into distinct quadrants in the principal component analysis (Figure 1). Ivacaftor exposure elicited contrasting responses: in Pa2713, ivacaftor alone or with colistin induced similar lipid A modifications, whereas in Pa1013, phosphoethanolamine lipid A profiles differed markedly between these conditions. Notably, checkerboard assays corroborated these findings, with Pa1013 exhibiting greater synergistic susceptibility to colistin plus ivacaftor than Pa2713.

### 2.4. Mouse Model of Acute Pneumonia

**Figure 2:**
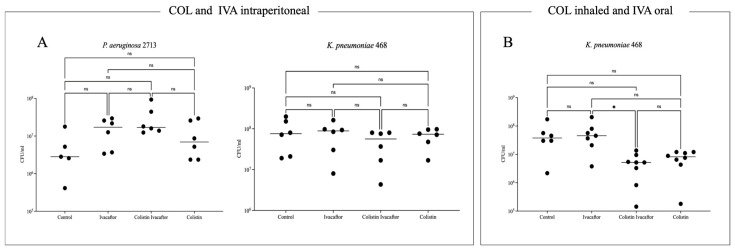
Bacterial count in the lungs in a murine model of acute pneumonia. First, we administered colistin sulfate and ivacaftor intraperitoneally (**A**). In a second phase, we administered ivacaftor orally and CMS intranasally (**B**). ns: not significative, * *p* ≤ 0.05.

The in vivo synergistic effects of colistin and ivacaftor were evaluated using colistin-resistant Pa2713 and Kp468 isolates in a murine model of acute pneumonia induced by intranasal inoculation. This mouse model of acute lung infection is highly suitable for short-term treatments and to avoid the extra challenge of biofilm formation [18]. In the initial experiment, colistimethate sodium (CMS) and ivacaftor were administered intraperitoneally at the time of infection and 6 h post-infection. At 24 h post-infection, mice exhibited a mean clinical score of 2 (Thomsen scale), with no significant differences in lung bacterial burden across treatment groups (Figure 2). A second experiment was conducted with Kp468, using inhaled CMS and oral ivacaftor. Lung bacterial quantification showed no differences compared to the control group, indicating a lack of in vivo synergy between ivacaftor and colistin. Consequently, the experiment with *P. aeruginosa* was not repeated for ethical reasons (Figure 2).

## 3. Discussion

Colistin, a critical last-resort antibiotic, retains activity against most multidrug-resistant Gram-negative bacteria. Despite prolonged use, colistin resistance remains uncommon, potentially due to deleterious mutations or other unknown factors. Resistance mechanisms vary by genus and lineage, involving chromosomal gene mutations affecting cell wall biosynthesis or plasmid-mediated *mcr* genes [15]. In subjects with CF, inhaled CMS is a standard therapy, yet resistance in CF-associated *P. aeruginosa* is rare, primarily linked to phosphoethanolamine or 4-amino-4-deoxy-L-arabinose (Ara4N) addition to lipid A via *eptA* or the *arn* and *pmr* operons [19,20].

The introduction of CFTR modulators has markedly reduced *P. aeruginosa* density in CF respiratory samples [3,4,5], prompting speculation about their antimicrobial activity, particularly for ivacaftor due to its quinolone-like structure [21]. Our findings confirm that the corrector lumacaftor lacks antimicrobial activity, consistent with prior reports [22]. Ivacaftor exhibits bacteriostatic effects against certain Gram-positive bacteria [23] and synergistic activity with antibiotics like ciprofloxacin [24] and polymyxin B [9].

This study is the first to report in vitro synergy between ivacaftor and colistin, reversing colistin resistance in both *P. aeruginosa* and *K. pneumoniae* clinical isolates. In colistin-susceptible isolates, ivacaftor reduced colistin MICs by 2–3-fold, while in resistant isolates, MIC reductions were more pronounced: 5–6-fold in *P. aeruginosa*, 4-fold in *K. pneumoniae*, and 4-fold in *mcr*-1 *E. coli*.

Colistin resistance often arises from phosphoethanolamine modifications to lipid A, neutralizing the membrane’s negative charge and reducing colistin binding. Our metabolomic analysis revealed that exposure to ivacaftor, colistin, or both induced strain-specific phosphoethanolamine alterations in two colistin-resistant *P. aeruginosa* isolates (Pa1013 and Pa2713). These findings suggest diverse lipid A modification strategies, complicating mechanistic analyses, especially given recent evidence of greater phosphoethanolamine diversity than previously recognized [25]. Consequently, the molecular basis of ivacaftor-colistin synergy remains unclear and may require strain-specific investigations.

Despite promising in vitro results, in vivo synergy was not observed in a murine acute pneumonia model using two administration routes (intraperitoneal and inhaled/oral). Although prior studies reported efficacy with intraperitoneal ivacaftor (25 mg/kg) and CMS [23] against *S. aureus*, our experiments showed no synergistic or additive effects, as evidenced by unchanged clinical scores and lung bacterial burdens. The inhaled/oral route, mimicking CF treatment [26], similarly yielded no differences for *K. pneumoniae*.

Several factors may explain these discrepancies. Experimental conditions, including growth media, colistin forms (sulfate vs. CMS), and administration routes, vary across studies and may influence outcomes. Optimal dosing, informed by checkerboard assays in this study and prior reports [9,23,27], is critical for synergy. The lack of in vivo reproducibility suggests limitations in the acute pneumonia model. Adequate drug concentrations at the infection site cannot be guaranteed, and alternative doses or prolonged treatments warrant exploration.

Chronic CF lung infections often involve bacterial biofilms, which colistin penetrates effectively in susceptible *P. aeruginosa* [28]. However, biofilms may alter drug interactions, and our model did not account for this. Additionally, *P. aeruginosa* lung infections in mice, especially acute lung models, tend to resolve spontaneously, potentially masking treatment effects [29]. A biofilm-based model, such as a wound infection setup [30], with extended treatment durations, could potentially better elucidate in vitro–in vivo discrepancies.

As a limitation of this work, we identified that the absence of synergy in vivo may reflect specific model constraints rather than a lack of clinical potential. Unknown factors, including biofilm dynamics and pharmacokinetics, cannot be ruled out. Future studies should explore biofilm models, optimized dosing, and chronic infection scenarios to validate ivacaftor-colistin synergy.

## 4. Materials and Methods

### 4.1. Bacterial Strains and Susceptibility Assays

Six colistin-susceptible (Pa9, Pa15, Pa205, Pa443, Pa647, Pa746) and two colistin-resistant (Pa1013, Pa2713) *P. aeruginosa* isolates were obtained from respiratory samples of unrelated subjects with CF. Additionally, two colistin-resistant *K. pneumoniae* isolates (Kp468 from a rectal swab, Kp931 from urine) were collected from intensive care unit (ICU)-admitted non-CF patients. Genomic DNA was sequenced using the Illumina MiSeq platform and deposited under BioProject accession PRJNA1150980. Genomes were assembled using VelvetOptimiser v2.2.5 and SPAdes v3.9.0, annotated with Prokka v1.14.6, and analyzed for single-nucleotide polymorphisms (SNPs) using Snippy v4.6.0. Colistin resistance-associated genes and mutations were identified using the Comprehensive Antibiotic Resistance Database (CARD) and manual inspection. Colistin-susceptible *P. aeruginosa* ATCC 27853 and *Escherichia coli* NCTC 13846 (*mcr-1*-positive) served as controls. The study protocol was approved by the Ethical Committee of University Hospital Ramón y Cajal (registration 372/13).

Ivacaftor and lumacaftor (Selleckchem, Houston, TX, USA) were dissolved in 50% dimethyl sulfoxide (DMSO). Ivacaftor stock solutions were prepared in 50% DMSO. The final concentration of DMSO in antimicrobial assays was 0.078%, and equivalent DMSO concentrations were included in control wells. Colistin sulfate (Sigma-Aldrich, Saint Louis, MO, USA) was prepared following the European Committee on Antimicrobial Susceptibility Testing (EUCAST) guidelines. Colistin minimum inhibitory concentrations (MICs) were determined using the ISO-standard broth microdilution method in cation-adjusted Mueller-Hinton broth (CAMHB; Difco, Beqaa, Lebanon) alone or with ivacaftor (16 mg/L), lumacaftor (16 mg/L), or ivacaftor-lumacaftor (16 mg/L each) in microtiter plates. Each strain was tested in three independent experiments, with MICs reported as the median. Checkerboard assays were performed for colistin-resistant strains, combining colistin and ivacaftor at concentrations ranging from 0.5 to 16 mg/L (two-fold serial dilutions) in CAMHB.

### 4.2. Metabolomic Analysis

Colistin-resistant isolates Pa1013, Pa2713 were cultured at 10^7^ CFU/mL in Brain Heart Infusion (BHI; Difco) broth at 37 °C with agitation for 4 h, with or without colistin (1 mg/L) and/or ivacaftor (16 mg/L). Experiments were conducted in 10 replicates, with three 1.5-mL aliquots collected per condition. Aliquots (n = 112, including controls) were centrifuged at 14,000× *g* for 2 min at 4 °C, and pellets were frozen in liquid nitrogen. Samples were analyzed at Fundación Medina (Granada, Spain) using liquid chromatography–high-resolution mass spectrometry (LC-HRMS). Pellets were reconstituted, transferred to analytical vials, and analyzed on an AB SCIEX TripleTOF 5600 quadrupole-time-of-flight mass spectrometer in positive ion mode, coupled to an Agilent 1290 LC system (Agilent Technologies, Santa Clara, CA, USA). Chromatographic separation was performed using a Waters Atlantis T3 HPLC column (C18, 2.1 × 150 mm, 3 µm; Waters Corporation, Milford, MA, USA) at 30 °C, with a 5-µL injection volume.

LC-HRMS data were processed using MarkerView software v1.2.1 (AB SCIEX, Concord, ON, Canada) for peak detection, alignment, and filtering, generating a data matrix of mass-to-charge ratio (*m*/*z*), retention time, and ion peak area. Statistical analysis included univariate and multivariate approaches. UVA used t-tests or ANOVA (*p* < 0.05, corrected for false discovery rate) to identify significant differences. Multivariate approach employed partial least squares-discriminant analysis, with variable importance in projection scores (>1) from the first five components used to select discriminant metabolites.

### 4.3. Murine Acute Pneumonia Model

Specific pathogen-free female BALB/c mice (9–11 weeks old; Janvier Labs, Le Genest Saint Isle, France) were housed in filtered cages with ad libitum access to standard food and water for at least 1 week to acclimatize. Experiments were approved by the Animal Experiments Inspectorate, Ministry of Food, Agriculture and Fisheries of Denmark (permit 2019-15-0201-00183). Colistin-resistant Pa2713 and Kp468 isolates were cultured overnight in BHI at 37 °C with agitation, adjusted to 10^8^ CFU/mL in saline, and verified by culture on modified Conradi–Drigalski plates (SSI Diagnostica 694, Hillerod, Denmark) before and after administration. Mice were anesthetized with 3% isoflurane (3 L/min, 50 s) and inoculated intranasally with 40 µL of bacterial suspension. Mice were randomized into four groups (n = 6 per group) receiving intraperitoneal colistimethate sodium (CMS; 50 mg/kg; Altan GES, Madrid, Spain), ivacaftor (25 mg/kg), CMS plus ivacaftor, or placebo (saline with DMSO) at 0 and 6 h post-infection. A second experiment with Kp468 used oral ivacaftor (60 mg/kg, 1 h pre-infection) and inhaled CMS (5 mg/kg, concurrent with infection).

Clinical condition was assessed 24 h post-infection by a blinded observer using the Thomsen scoring system [18], ranging from 0 (unaffected) to 5 (dead). Mice were euthanized with intraperitoneal fluanisone and midazolam, and lungs were aseptically removed, weighed, and homogenized in 4 mL of 0.9% NaCl with glass beads using a TissueLyser II (Qiagen, Hilden, Germany) at 30 Hz for 20 min. Homogenates were serially diluted, plated on modified Conradi–Drigalski plates, and incubated at 37 °C overnight for bacterial enumeration. Colony-forming units (CFU) per lung were calculated by multiplying colony counts by the dilution factor. Lung bacterial burdens were compared using the Kruskal–Wallis test in GraphPad Prism v11 (GraphPad Software, San Diego, CA, USA), with *p* ≤ 0.05 considered statistically significant.

## 5. Conclusions

We report novel in vitro synergy between ivacaftor and colistin, restoring colistin susceptibility in resistant CF-*P. aeruginosa* and *K. pneumoniae* strains. This effect may involve strain-specific lipid A phosphoethanolamine modifications to Lipid A in their LPS. However, the lack of in vivo confirmation in an acute pneumonia model limits immediate clinical application.

## Figures and Tables

**Table 1:** Minimal inhibitory concentration for colistin alone or in combination with 16 mg/L of ivacaftor and/or 16 mg/L of lumacaftor.

Isolate (Sequence Type, ST)	MIC (mg/L)			
	Colistin	Colistin Ivacaftor	Colistin Lumacaftor	Colistin Ivacaftor Lumacaftor
*P. aeruginosa*				
Pa9 (ST1109)	2	0.25	1	0.25
Pa15 (ST851)	2	0.5	1	0.5
Pa205 (ST412)	0.5	0.12	0.5	0.25
Pa443 (ST575)	0.5	0.12	0.5	0.25
Pa647 (ST575)	1	0.25	0.5	0.5
Pa746 (ST575)	0.25	0.06	0.25	0.06
Pa1013 (ST3159)	≥64	1	8	1
Pa2713 (ST238)	64	2	8	2
Pa ATCC27853	1	0.5	1	0.5
*K. pneumoniae*				
Kp468 (ST340)	≥64	4	≥64	4
Kp931 (ST11)	32	2	32	2
*E. coli mcr*-1	4	<0.25	4	<0.25

**Table 2:** Checkerboard assay expressing colistin MIC value (mg/L) in the presence of increasing concentrations of ivacaftor (from 0 to 16 mg/L). Bold values indicate those concentrations of ivacaftor at which colistin MIC was halved. The accepted accuracy is ±1 dilution.

Bacterial Strains	Original Colistin MIC (mg/L)	Ivacaftor (mg/L)						
		0	0.5	1	2	4	8	16
Pa9	2	2	2	2	**1**	0.5	0.5	0.25
Pa15	2	2	2	**1**	0.5	0.5	0.5	0.5
Pa205	0.5	0.5	0.5	**0.25**	0.12	0.12	0.12	0.12
Pa443	0.5	0.5	0.5	**0.25**	0.25	0.25	0.25	0.12
Pa647	1	1	1	1	**0.12**	0.12	0.12	0.12
Pa746	0.25	0.25	0.25	0.25	**0.12**	0.06	0.06	0.06
Pa1013	≥64	≥64	64	**8**	4	2	2	1
Pa2713	64	64	64	**32**	8	4	4	2
Kp468	≥64	≥64		**4**				
Kp931	32	32		**2**				

**Table 3:** Identification of the 6 metabolomic peaks with significant differential levels in the 4 studied conditions: control without supplementation, supplementation with ivacaftor (IVA), with colistin (COL), and ivacaftor plus colistin (IVA + COL).

Formula	Identification	Significantly Increased in Condition (Strain and *p* Value):	Fold Change (*p* Value)
C35H70NO8P	1-Myristoyl-2-palmitoyl-sn-glycero-3-phosphoethanolamine (16:0/14:0)	IVA vs. COL + IVA (Pa1013)	14.85 (1.2 × 10^−12^)
		Control vs. COL + IVA (Pa1013)	11.18 (7.4 × 10^−14^)
C37H72NO8P	1-palmitoyl-2-palmitoleoyl-sn-glycero-3-phosphoethanolamine (16:0/16:1)	IVA vs. COL + IVA (Pa1013)	16.80 (9.5 × 10^−12)^
		COL + IVA vs. IVA (Pa2713)	0.38 1.5 × 10^−8^)
		Control vs. COL + IVA (Pa1013)	9.88 (2.1 × 10^−11^)
		COL + IVA vs. CONTROL (Pa2713)	0.45 (6.4 × 10^−7^)
C37H74NO8P	1,2-Dipalmitoyl-sn-glycero-3-phosphoethanolamine (16:0/16:0)	IVA vs. COL + IVA (Pa1013)	17.84 (2.7 × 10^−12^)
		COL + IVA vs. IVA (Pa2713)	0.59 (2.9 × 10^−7^)
		Control vs. COL + IVA (Pa1013)	12.98 (2.5 × 10^−13^)
C39H76NO8P	1-palmitoyl-2-oleoyl-sn-glycero-3-phosphoethanolamine (16:0/18:1)	IVA vs. COL +IVA (Pa1013)	8.76 (5.3 × 10^−11^)
		COL + IVA vs. IVA (Pa2713)	0.36 (2.9 × 10^−9^)
		Control vs. COL + IVA (Pa1013)	5.96 (4.7 × 10^−11^)
		COL + IVA vs. CONTROL (Pa2713)	0.36 (6.0 × 10^−9^)
C41H78NO8P	1-(11Z-Octadecenoyl)-2-(9Z-octadecenoyl)-sn-glycero-3-phosphoethanolamine (18:1/18:1)	COL + IVA vs. IVA (Pa2713)	0.50 (1.0 × 10^−7^)
		COL + IVA vs. CONTROL (Pa2713)	0.59 (3.2 × 10^−9^)
C16H21NO	2-Heptyl-4-hydroxyquinoline	Control vs. IVA (Pa1013)	0.14 (4.8 × 10^−9^)
		Control vs. IVA (Pa2713)	0.07 (1.8 × 10^−8^)
		Control vs. COL (Pa1013)	0.27 (4.3 × 10^−7^)

## Data Availability

Whole-genome sequence data for the clinical isolates used in this study are available from the NCBI BioProject database under accession number PRJNA1150980. Other raw data or additional information will be provided by the corresponding author upon reasonable request.
